# CD55 is over-expressed in the tumour environment

**DOI:** 10.1054/bjoc.2000.1570

**Published:** 2001-01-01

**Authors:** L Li, I Spendlove, J Morgan, L G Durrant

**Affiliations:** CRC Academic Unit of Clinical Oncology, University of Nottingham, City Hospital, Hucknall Road, Nottingham, NG5 1PB, UK

**Keywords:** CD55 (DAF), tumour expression, endothelium, stroma, vascular endothelial growth factor (VEGF)

## Abstract

CD55 is a protein that protects cells from complement-mediated attack. 791Tgp72 is an antigen which has been used succesfully as a target for both tumour imaging and cancer vaccines. 791Tgp72 has recently been identified as CD55. Quantitative expression of CD55 in the tumour environment was therefore studied. Tumour cells showed a 4–100-fold increase in CD55 cell surface expression when compared to normal cells. Immunohistochemical staining of colorectal tumours also revealed high expression of CD55 in the stroma. To examine the source of this stromal CD55 the ability of both epithelial cells and endothelial cells to produce extracellular CD55 was measured. Tumour cell lines deposit CD55 into their extracellular matrix (ECM) in direct proportion to their cell surface expression. In contrast the ECM from HUVEC cells contained large amounts of CD55 despite expressing low levels of CD55 on their cell surface. Furthermore expression of CD55 on HUVEC cells was increased by exposure to VEGF. Although it remains unclear why CD55 is upregulated in the tumour environment its high level of expression on tumour cells and associated endothelium may explain why it is a good target for both imaging and immunotherapy. © 2001 Cancer Research Campaign http://www.bjcancer.com


British Journal of Cancer (2001) 84(1), 80–86
© 2001 Cancer Research Campaign
doi: 10.1054/ bjoc.2000.1570, available online at http://www.idealibrary.com on IDEAL®

### CD55 is over-expressed in the tumour environment

**L Li, I Spendlove, J Morgan and LG Durrant**

CRC Academic Unit of Clinical Oncology, University of Nottingham, City Hospital, Hucknall Road, Nottingham, NG5 1PB, UK

**Summary** CD55 is a protein that protects cells from complement-mediated attack. 791Tgp72 is an antigen which has been used successfully as a target for both tumour imaging and cancer vaccines. 791Tgp72 has recently been identified as CD55. Quantitative expression of CD55 in the tumour environment was therefore studied. Tumour cells showed a 4–100-fold increase in CD55 cell surface expression when compared to normal cells. Immunohistochemical staining of colorectal tumours also revealed high expression of CD55 in the stroma. To examine the source of this stromal CD55 the ability of both epithelial cells and endothelial cells to produce extracellular CD55 was measured. Tumour cell lines deposit CD55 into their extracellular matrix (ECM) in direct proportion to their cell surface expression. In contrast the ECM from HUVEC cells contained large amounts of CD55 despite expressing low levels of CD55 on their cell surface. Furthermore expression of CD55 on HUVEC cells was increased by exposure to VEGF. Although it remains unclear why CD55 is upregulated in the tumour environment its high level of expression on tumour cells and associated endothelium may explain why it is a good target for both imaging and immunotherapy. © 2001 Cancer Research Campaign http://www.bjcancer.com

**Keywords:** CD55 (DAF), tumour expression; endothelium; stroma; vascular endothelial growth factor (VEGF)

CD55 is expressed on cells to protect them from complement attack. It is therefore present on all cells that are exposed to complement including red blood cells, leukocytes, endothelial cells and epithelial cells. CD55 binds C3 convertases from both the classical and alternative complement pathways preventing C3b deposition and inhibiting the downstream assembly of the membrane attack complex. CD55 is a GPI-anchored protein composed of 4 contiguous short consensus repeat (SCR) domains and a Serine/Threonine (S/T) rich region proximal to the cell surface (Nicholson-Weller and Wang, 1994).

791Tgp72 is a tumour associated antigen which has been used successfully as a target for tumour imaging with the mouse monoclonal antibody 791T/36. Osteosarcomas, ovarian, gastric and colorectal tumours have been successfully imaged with lesions as small as 1 $cm^3$ being detected (Farrands et al, 1982, 1983; Armitage et al, 1985). A human monoclonal anti-idiotypic antibody 105AD7 was shown to mimic the 791Tgp72 antigen (Austin et al, 1989) and stimulate anti-tumour T cell responses in cancer patients without any associated toxicity. A phase I study in advanced colorectal cancer patients demonstrated enhanced plasma IL-2 production, conversion of both CD4 and CD8 cells from CD45 RA (naïve) to CD45 RO (memory) cells (Durrant et al, 2000) and in vitro blastogenesis responses to tumour cells expressing 791Tgp72 (Denton et al, 1994). In a neoadjuvant study patients were immunized pre-operatively and boosted following resection of their colorectal tumours. Tumours from the immunized group showed enhanced infiltration of CD4 and CD56 cells compared to unimmunized tumours. Infiltration of CD56 cells correlated well with enhanced NK activity (Durrant et al, 1999). There was also enhanced killing of autologous tumour cells which was unrelated to NK killing (Durrant et al, 1994) and a three-fold increase in tumour cell apoptosis.

791Tgp72 has recently been characterized as CD55 (Spendlove et al, 1999) and it was of interest to establish why CD55 was a target for tumour immunotherapy, despite its apparent ubiquitous expression. Expression of CD55 in the tumour environment was therefore studied.

*Received 27 September 1999*
*Revised 3 February 2000*
*Accepted 15 March 2000*

*Correspondence to:* LG Durrant

### MATERIALS AND METHODS

### Cells

791T and MG63 are osteosarcoma cell lines. Colo205, C170, SW480, PT01, HT29 are all colorectal cell lines whereas MKN45 and MKN45K are both gastric cell lines. All cell lines were grown in RPMI (Gibco, BRL, Paisley, UK) supplemented with 10% heat inactivated fetal calf serum. Red blood cells and lymphocytes were isolated from the same peripheral blood sample by centrifugation through Ficoll. Leukocytes were collected at the Ficoll/serum interface and red blood cells were pelleted to the bottom of the tube. Monocytes were obtained following overnight plastic adherence of Ficoll-purified leukocytes. Human umbilical vein endothelial cells (HUVEC) were isolated by the method of Jaffe (Jaffe et al, 1972). Endothelial cells were maintained in growth medium consisting 1:1 of M199 and Ham F12 nutrient mixture (Sigma, Dorset, UK) supplemented with 0.014 M N-(2-hydroxyethyl) piperazine-N'-(2-hydroxy-propane) sulphonic acid (HEPES), 0.15% sodium bicarbonate, 2 mM L-glutamine, 10 U $ml^{-1}$ penicillin/10 U $ml^{-1}$ streptomycin (Sigma), 30% (v/v) fetal calf serum (Gibco-BRL), 15 U $ml^{-1}$ heparin and 40 U $ml^{-1}$ endothelial cell growth supplement (Boehringer Mannheim, Lewes, UK) and passed at a 1:3 split ratio onto 0.2% gelatine-coated flasks. Upregulation of CD55 by either tumour-conditioned medium or VEGF was demonstrated by splitting cells 1:3 in 1:1 mix of fresh and tumour-conditioned medium or VEGF (10–300 μg ml$^{-1}$: Zeneca, Alderley Park, UK) respectively for 48 h prior to harvesting the HUVECs. Tumour-conditioned media was derived from the MDA 231 breast cancer cell line. These were grown to confluence and the media replaced with serum free M199:Hams F12 medium. Following 24 h incubation the resultant conditioned media was filtered for use with the HUVEC. Fresh media was used to culture the control HUVECs. Freshly resected tumours were finely minced and dissociated in collagenase (0.05%; Boehringer Mannheim) and DNase (0.1%; Boehringer Mannheim) for 1 h at 37˚C.

### Monoclonal antibodies

Monoclonal antibodies 791T/36 (IgG2b anti-791Tgp72; Embleton et al, 1981), BRIC 216 (IgG1 anti-SCR 3 of CD55; Tate et al, 1989), BRIC 220 (IgG1 anti-SCR 1 of CD55; Tate et al, 1989), BRIC 110 (IgG1 anti-SCR 2 of CD55; Spring et al, 1987; Coyne et al, 1992) have been reported previously. BRIC 229 and E4.3 (Sparrow and Mckenzie, 1983) recognize CD59 and CD46 respectively. The BRIC antibodies were purchased from the Blood Group Reference laboratory (Bristol, UK). Normal mouse IgG (Sigma, Dorset, UK) and 708 monoclonal antibody (IgG2b) was used as negative control antibodies.

### Indirect immunofluorescence

Cell lines were incubated with monoclonal antibody for 1 h at 4˚C. Cells were washed twice in fresh media containing 1% FCS and then incubated with FITC conjugated rabbit anti-mouse (1/100, Dako, High Wycombe, UK). Cells were analysed on a FACSscan (Becton Dickinson, Sunnyvale CA, USA). Fluorescein fluorescence was excited at 488 nm and collected via a 10 nm band with band pass filter centred at 515 nm after adjustment for standard conditions using fluorochrome-labelled latex beads. Fluorescence intensity expressed as mean linear fluorescence (MLF) was calculated by multiplying the contents of each channel by its channel number and dividing by the total number of cells in the distribution.

### Immunohistochemistry

Sections (5 μm) were cut from cryopreserved tumour blocks, air dried overnight and rehydrated with Tris buffered saline (TBS). Sections were preincubated with 100 μl of 20% rabbit serum for 20 min prior to the addition of 100 μl of the primary antibodies (791T/36, 216, 220 or 110) to consecutive sections of tumour. After 1 h the slides were washed and 100 μl of rabbit anti-mouse conjugated to horseradish peroxidase (Sigma) was added in TBS containing 4% human serum. Staining was developed with 3,3'-diaminobenzidine-tetrahydrochloride (DAB-Sigma). After 10 min the slides were transferred to a 0.5% copper sulphate bath for 10 min and then stained with haematoxylin. Slides were then dehydrated and mounted.

### Western blotting

Cells were washed twice with PBS and solubilized in 1% octyl-glucoside (Sigma) in lysis buffer (20 mM Tris.HCl pH 8.0, 150 mM NaCl). After half an hour at 4˚C, the samples were centrifuged at 13 000 rpm for 10 minutes. The supernatant was removed to a clean tube and centrifuged again at 100 000 ***g*** for 30 min. Cell lysates (from between $5 \times 10^5$ and $1 \times 10^7$ according to cell type) were loaded on to SDS-PAGE under non-reducing conditions. Proteins were transferred to nitrocellulose membrane and blocked with PBS containing BSA (1%) for 1 h at room temperature. After two washes with PBS-Tween (0.1%), primary antibody was added for 1 h at room temperature. The blots were washed twice and rabbit anti-mouse HRP-conjugate (Dako) diluted 1:1000 was added. Following 1 h incubation and 3 washes, the blots were developed by enhanced chemilluminescence (ECL) system (Amersham-Pharmacia Biotech, Bucks, UK).

### Preparation and screening of extracellular matrix (ECM) for CD55

CD55 was measured in ECM as previously described (Hindmarsh and Marks, 1998). HUVEC and tumour cells were cultured on 96-well flat-bottom plates and maintained for up to 7 days post-confluence by replacement of culture media every 1–3 days. Post-confluent cells were removed using 0.02% EDTA in PBS at 37˚C, leaving the ECM attached to the plate. ECM was washed 3 times with PBS and used immediately. After blocking with 1% BSA for 1 h, 791T/36 monoclonal antibody or the IgG2b control antibody 708 was added to the plates for 1 h prior to washing twice with PBS-Tween 20 (0.05%). The plates were incubated with conjugated rabbit anti-mouse horseradish peroxidase (Dako) diluted 1:1000 for a further 1 h. Plates were washed 3–4 times in PBS with Tween 20 (0.05%) and developed with ABTS (Sigma). Colour development was measured after 15–30 min at 405 nm.

### RESULTS

Expression of CD55 was studied on a panel of tumour and normal cells by indirect immunofluorescence and flow cytometric analysis (Table 1). Cells were stained with the anti-tumour monoclonal antibody 791T/36, which binds to domains 1 and 2 of CD55, and BRIC 220, 110 and 216 monoclonal antibodies, which bind to CD55 SCR domains 1, 2 and 3 respectively. The normal cells in this study have low level of expression of CD55. In contrast, however, the tumour cell lines showed a 5–100-fold increase in expression of CD55. The only exception was Colo205 that failed to show any increased expression of CD55. The colorectal cell lines expressed 5–18 fold more than the normal gastrointestinal cells. The gastric cell lines showed an even greater increase in expression (35–53-fold) and the osteosarcoma cell line (791T) showed the strongest expression (>100 fold). The level of expression was also confirmed using the well-characterized anti-CD55 antibodies BRIC 110, 216 and 220, which showed slight variations from the levels obtained with 791T/36. By comparison total cellular pools of CD55 were analysed by SDS-PAGE and Western blotting using the monoclonal antibody BRIC 216 (Fig. 1). $1–2 \times 10^7$ red blood cells or T-cells were required to obtain a visible band, compared to $1 \times 10^5$ tumour cells that gave a band of approximately 4-fold greater intensity. The overall level of expression correlated well with the immunofluorescence results with 791T > MKN45K > HT29 > C170 > HUVEC > lymphocytes = red blood cells, suggesting that most of the CD55 pool was membrane associated. Slight variations in size were observed among CD55 isolated from various cells. 791T, HT29, C170, HUVEC and lymphocytes express a 72 kDa form, whereas the band in MKN45 is slightly larger and the band in red blood cells

© 2001 Cancer Research Campaign 

**Table 1** Expression of CD55 on normal cells and tumour cell lines

| Cells[a] | Origin | Staining (MLF[b]) with mouse monoclonal antibodies recognizing: | | | | | | Mouse IgG[c] | T:N ratio[d] |
|---|---|---|---|---|---|---|---|---|---|
| | | CD55 | | | | CD46 | CD59 | | |
| | | 791T/36 | 110 | 216 | 220 | E4.3 | 229 | | |
| *Tumour cell lines* | | | | | | | | | |
| 791T | Osteosarcoma | 2631 | 2005 | 2376 | 2160 | 540 | 3982 | 40 | 104 |
| C170 | Colorectal | 412 | 246 | 302 | 271 | 650 | 1700 | 17 | 16 |
| HT29 | Colorectal | 495 | 312 | 388 | 339 | 778 | 852 | 30 | 18 |
| SW480 | Colorectal | 160 | 85 | 88 | 85 | ND | ND | 25 | 5 |
| Colo205 | Colorectal | 63 | 34 | 28 | 27 | 912 | 356 | 34 | 1 |
| PTO1 | Colorectal | 167 | 190 | 161 | 166 | ND | ND | 14 | 6 |
| MKN45K | Gastric | 995 | 731 | 1022 | 728 | 800 | 1900 | 121 | 35 |
| MKN45 | Gastric | 1387 | 1355 | 1317 | 1444 | ND | ND | 70 | 53 |
| *Normal cells* | | | | | | | | | |
| | Red blood cells | 60 | 53 | 94 | 101 | ND | ND | 27 | 1 |
| | HUVECs | 146 | 112 | 130 | 101 | 66 | ND | 56 | 4 |
| | Lymphocytes | 60 | 51 | 54 | 51 | ND | ND | 4 | 2 |
| | Monocytes | 64 | ND | 67 | ND | 101 | 396 | 8 | 2 |
| | Gastrointestinal epithelial cells | 35 | ND | ND | ND | ND | ND | 10 | 1 |

[a] Cells were harvested, washed, stained by indirect immunofluorescence and analysed by flow cytometry. [b] Results are expressed as mean linear fluorescence. [c] Polyclonal mouse IgG was used as a negative control for all antibodies. MLF tumour cell line 791T/36-MLF normal mouse. [d] T:N ratio is defined as MLF GI epithelial cells 791T/36-MLF normal mouse. ND-Not determined.

smaller. 791T cells and to a lesser extent C170 cells also express a second 66 kDa isoform.

To determine if the over-expression of CD55 observed on established cell lines could also be seen in tumours in situ, 17 freshly resected colorectal (Figure 2A) and 18 gastric tumours (Figure 2B) were disaggregated and stained with the anti-CD55 monoclonal antibody 791T/36 for analysis by FACS. For comparison normal mucosa (Figure 2C), obtained from the operation resection margins and 4 resected adenomas (Figure 2D) were also disaggregated and stained with 791T/36. Similar levels of CD55 expression were observed on the primary colorectal tumours as were seen on the established cell lines. One colorectal tumour failed to express CD55, whereas a further two showed similar levels to normal gastrointestinal cells. 80% of the colorectal tumours showed 4–50-fold increases in CD55 expression. The gastric tumour cells showed very similar expression levels to the colorectal tumours. 83% had a 6–60-fold increase in CD55 expression, with one negative and two displaying normal levels of expression. The adenomas had similar levels of CD55 to the normal gastrointestinal mucosal samples.

A possible reason for over-expression of a protein may be to compensate for a deficiency of another functionally related protein. In the complement control protein family there are two other membrane associated regulators of complement, CD46 (Membrane Cofactor Protein, MCP) and CD59 (protectin). Analysis of the tumour cells for expression of CD46 and CD59 showed elevated levels of both molecules compared to normal cells (Table 1).

Immunohistochemical staining of colorectal tumours shows very strong stromal staining with the anti-CD55 monoclonal antibodies (Fig. 3). To identify the source of this extracellular CD55 both tumour and endothelial cells were assessed for their ability to secrete CD55. The supermatant from both cell types was used in

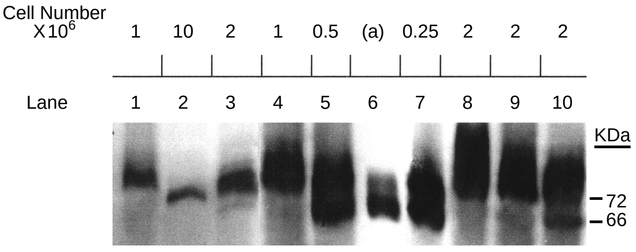

**Figure 1** Western blot analysis of CD55 expression on cells. The cell lysates were analysed by non-reducing SDS-PAGE and transferred to nitrocellulose membrane. The blots were incubated with the anti-CD55 monoclonal antibody BRIC 216 diluted 1:500, then with rabbit anti-mouse conjugated HRPO (1:1000) and developed using the ECL system. Lanes; 1) HUVEC ($1 \times 10^6$); 2) Erythrocytes ($1 \times 10^7$); 3) T cells ($2 \times 10^6$); 4) MG63 cells ($1 \times 10^6$); 5) 791T cells ($5 \times 10^5$); 6) (a) Approximately 150 ng of CD55 affinity purified from 791T cells; 7) 791T cells ($0.25 \times 10^5$); 8) MKN45K cells ($2 \times 10^6$); 9) HT29 cells ($2 \times 10^6$). 10) C170 cells ($2 \times 10^6$)

ELISA to measure the presence of soluble CD55. Neither the tumour cell lines nor the endothelial cells were shown to secrete significant amounts of CD55 (data not shown). In contrast all tumour cells deposited CD55 into their ECM and the level appeared proportional to their cell surface expression. In contrast the extracellular matrix from HUVEC contained almost as much CD55 as 791T extracellular matrix despite expressing 28 fold less CD55 (HUVEC, MLF 90 and 791T, MLF 2589) on their cell surface (Fig. 4).

The growth characteristics of endothelial cells are known to be affected by soluble growth factors in the tumour environment. To test the effect of tumours on endothelial expression of CD55, HUVECs were cultured in tumour-conditioned media (TCM). Figure 5A shows 2–4 fold increases in CD55 cell surface expression when HUVEC were grown in TCM from the breast cell line

*© 2001 Cancer Research Campaign*

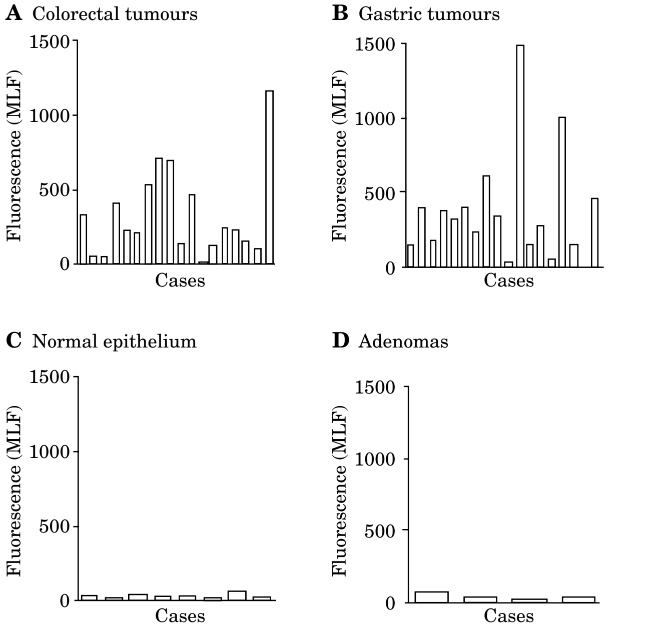

**Figure 2** Freshly isolated (**A**) colorectal tumour, (**B**) gastric tumour, (**C**) normal epithelium and (**D**) adenomas were disaggregated by collagenase treatment, stained by indirect immunofluorescence with 791T/36 monoclonal antibody and analysed by flow cytometry. Results are expressed as the mean linear fluorescence per tumour case

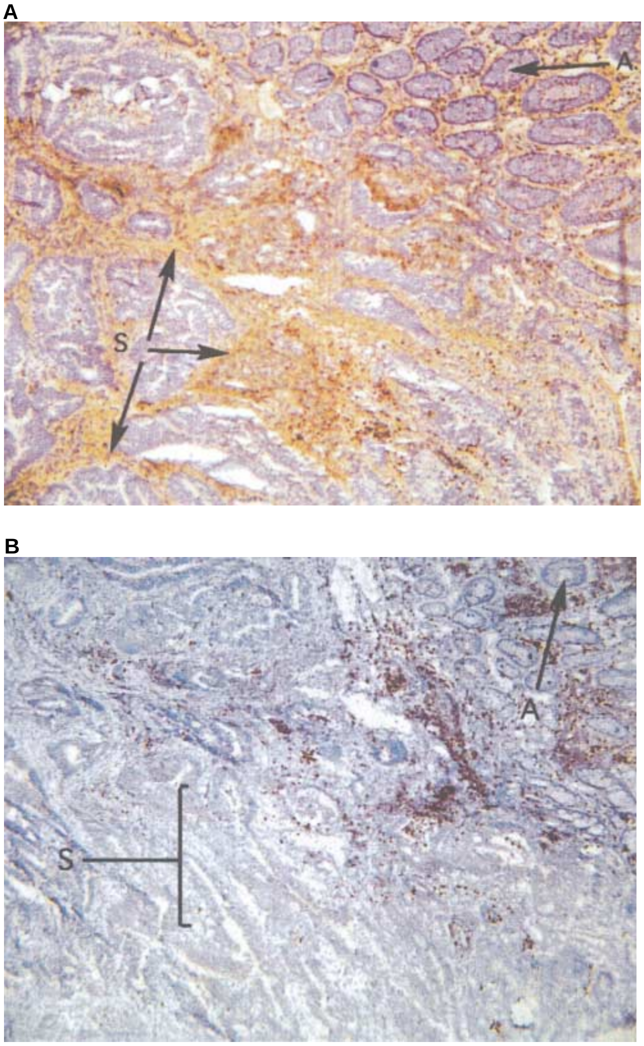

**Figure 3** Immunohistochemical staining of a colon tumour. Tumour sections were stained with (**A**) 216 monoclonal antibody and (**B**) normal mouse IgG. S defines tumour stroma and A adjacent mucosa

MDA 231. This cell line is known to be angiogenic and secretes high levels of VEGF (results not shown). The expression of CD55 in response to varying doses of VEGF was therefore studied (Figure 5B). CD55 expression doubled with as little as 30 ng/ml of VEGF. Higher doses only showed a slight further increase.

### DISCUSSION

CD55 has recently been shown to be the target for the tumour imaging monoclonal antibody 791T/36 and for the colorectal cancer vaccine, 105AD7. Cloning and sequencing of CD55 from tumour cells revealed that there were no mutations that would account for it being a specific target antigen on tumour cells (Spendlove et al, 1999). The relative levels of CD55 on tumour and normal cells were therefore studied.

All the normal cells in this study (endothelial cells, blood cells and gastrointestinal epithelial cells) expressed low levels of CD55 as determined by both immunofluorescence and Western blotting with a panel of anti-CD55 monoclonal antibodies. In contrast the majority of tumours over-expressed this antigen. The degree of over-expression varied widely with some tumours showing modest over-expression (2–10 fold) and others showing very high levels (>50 fold increase over normal cells). Similar results were obtained with established cell lines or tumour cells released from freshly resected tumours. Antibodies recognizing different domains of CD55 showed slightly different levels of binding to cells derived from different tissues. This may be related to the levels of glycosylation of CD55 in different tissues, which may affect the accessibility of each antibody. The high level of expression of CD55 by tumours has been noted in a number of other studies using immunohistochemical staining to monitor expression levels (Niehans et al, 1996). Furthermore this elevated level of expression is an independent indicator of prognosis with tumours that over-express CD55 progressing more rapidly (Buckley et al, 1996).

It has been proposed that over-expression of one of the complement inhibitory proteins (CIPs) may be a compensatory mechanism for the loss of another. The most notable variation in expression levels of the CIPs is in breast tumours assayed by immunohistochemistry where they demonstrated variable loss of one or more inhibitors (Niehans et al, 1996). In the gastric and colorectal tumour cell lines in this study all 3 CIPs, CD55, CD46 and CD59, were elevated.

In this study only two primary tumours failed to express CD55. Loss of CD55 expression is an infrequent occurrence in tumours with the notable exception of leukaemias. Here the loss of expression has been attributed to the loss of ability to synthesize a GPI-anchor and abnormal transcriptional regulation (Hatanaka et al, 1996). The absence of CD55 may leave cells vulnerable to complement-mediated lysis. However, the elevated levels of CD46 and CD59 observed on these cells should compensate for the absence of CD55.

Interestingly, immunohistochemistry of colorectal tumours with anti-CD55 monoclonal antibodies shows intense staining of the extracellular matrix. Both tumour and endothelial cells contribute to the extended stromal matrix within tumours. Previous studies have shown that both endothelial cells and the HeLa cell line can

© 2001 Cancer Research Campaign

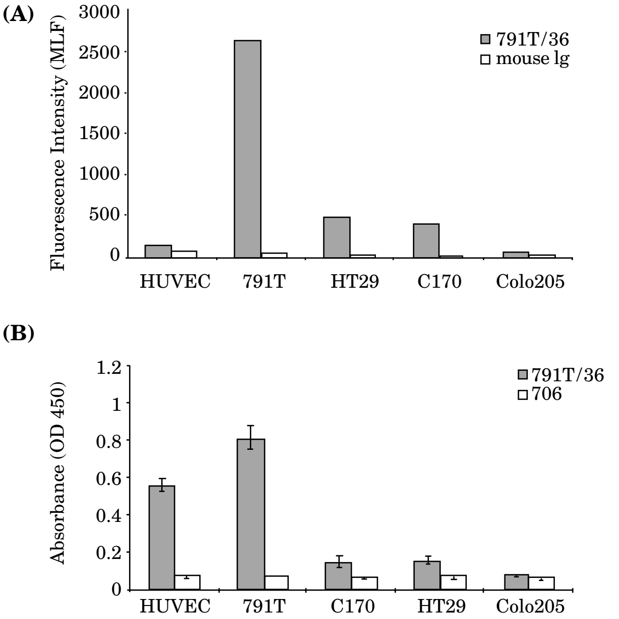

**Figure 4** Expression and deposition in the extracellular matrix of CD55 by tumour and endothelial cells. Tumour and endothelial cells were grown to confluence, removed from the culture wells by treatment with EDTA and both the cells and the extracellular matrix (ECM) were assayed for CD55. (**A**) Cells were measured by indirect immunofluorescence with 791T/36 and analysed by flow cytometry. Results are expressed as mean linear fluorescence. Normal mouse IgG was used as a negative control. (**B**) The extracellular matrix was assessed by 791T/36 ELISA. Monoclonal antibody 708 was used as a negative control

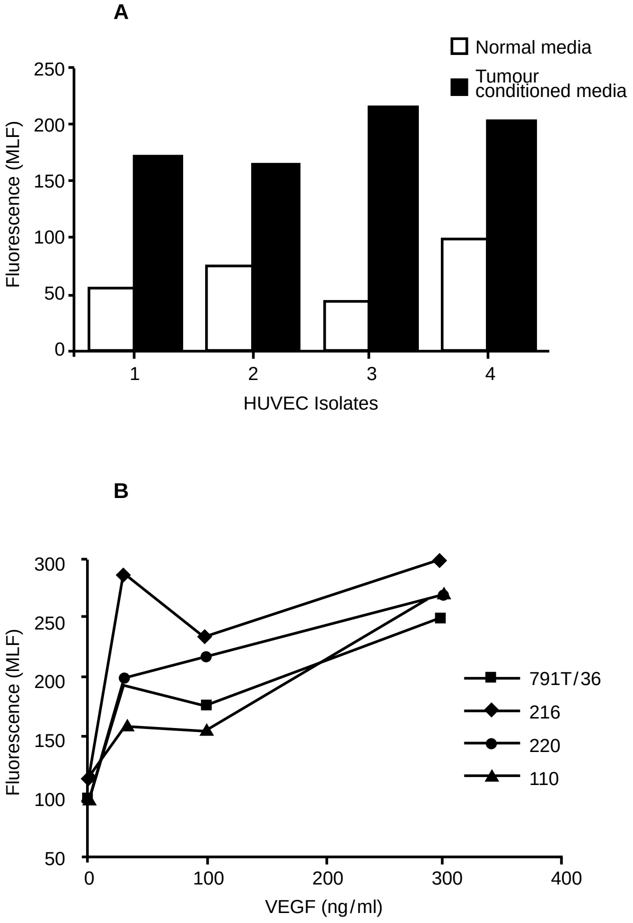

**Figure 5** The effect of (**A**) tumour-conditioned media and CD55 expression on HUVECs derived from 4 different donors. Cells were cultured in the presence (filled boxes) or absence (open boxes) of tumour-conditioned media for 24 hours. The cells were then assayed by indirect immunofluorescence using 791T/36 and analysed by flow cytometry. (**B**) Represents a single donor endothelial cell preparation treated with varying concentrations of VEGF (0–300 ng $ml^{-1}$). The cells were then assayed by indirect immunofluorescence using four anti CD55 antibodies. Results are expressed as mean linear fluorescence release CD55 into their ECM (Hindmarsh and Marks, 1998). This study has extended this observation to include osteosarcoma and colorectal cell lines. Furthermore it has shown that the amount of CD55 deposited into the ECM by tumour cells was proportional to cell surface expression. However, in endothelial cells, although the cell surface expression was similar to normal epithelia, the level of CD55 deposited into the ECM was closer to the levels deposited by the highest expressing tumour cell lines. Furthermore CD55 expression on endothelial cells was upregulated in the presence of both VEGF and tumour-conditioned media. Similar increases in expression of CD55 have also been reported with TNF-α, IL-1β, IL-4 and hypoxia (Collard et al, 1997; Hindmarsh and Marks 1998). Our data suggests that the tumour environment causes upregulation of CD55 on tumour-associated endothelium. Indeed Niehans et al (1996) observed high endothelial expression of CD55 in human cancers. The mechanism of CD55 deposition into the ECM is unclear. Previous studies have shown that HT29 cells can directly secrete CD55 without incorporating it into their cell membrane (Nasu et al, 1998). In contrast other studies have shown that tumour cells can flip phospholipase from the internal to the external membrane surface where it can specifically cleave GPI-anchored proteins. However, both of these mechanisms would also result in release of CD55 into culture supernatant. In this study only very low levels of CD55 could be detected in supernatant suggesting an alternative mechanism of CD55 deposition into ECM was taking place. Perhaps CD55 is covalently linked to cellular matrix proteins before it is exported from the cell? Whatever the mechanism of CD55 deposition into ECM it appears to remain functionally active as Hela derived ECM can efficiently deactivate complement (Hindmarsh and Marks, 1998).

It remains unclear as to why the tumour environment is such a rich source of CD55 when normal cells are protected from complement with more modest levels. This may be related to other emerging functions of CD55. It has recently been shown that CD55, like CD59, can act as a costimulator in submitogenically activated T-cells, resulting in secretion of IL-2 and cell proliferation (Tosello et al, 1998). Alternatively CD55 has recently been shown to be the ligand for CD97, an early activation marker expressed by activated leukocytes (Hamann et al, 1996). This raises the possibility that there may be epithelial leukocyte cross talk.

Numerous studies in patients with osteosarcoma, breast, ovarian and colorectal cancer have shown that radiolabelled 791T/36 monoclonal antibody localizes well in 70% of primary and metastatic lesions (Armitage et al, 1985). This study shows that 791T/36 could be localizing within the tumour either by binding to CD55 on tumour cells or within the stroma. Indeed autoradiography of resected tumours from patients imaged with radiolabelled 791T/36 showed intense stromal localization of the antibody (Armitage et al, 1984). Similarly over-expression of CD55 by

 © 2001 Cancer Research Campaign tumours makes it a good potential target for T cells. Other studies have shown that over-expressed self-antigens can be effective targets for cancer vaccines. When cytotoxic T lymphocytes (CTLs) specific to wild type p53 peptides were transferred to p53-bearing nude mice they caused complete and permanent tumour eradication. Importantly, this occurred in the absence of any demonstrable damage to normal tissue suggesting wild type p53-specific CTLs could discriminate between p53 over-expressing tumour cells and normal tissue (Vierboom et al, 1997). Similarly immunization with telomerase caused stimulation of CTLs that killed leukaemia cells expressing high levels of telomerase but failed to kill haematopoietic stem cells that expressed more modest levels of this enzyme (Vonderheide et al, 1999). This selective killing is thought to be related to the ability of T cells to count peptides associated with MHC (Lanzavecchia et al, 1999). The level of MHC occupancy of a normally expressed antigen is insufficient to allow effector T-cell triggering. However, increased antigen expression on tumours can raise the level of MHC occupancy to the threshold for T cell triggering. The relative levels of antigen expression between tumour and normal cells will depend upon peptide/MHC/TCR affinities. Levels of expression of CD55 on tumours compared to that on normal tissue make this molecule a good target for immunotherapy. This is evident in trials in colorectal cancer patients with the anti-idiotypic antibody that mimics CD55 (Denton et al, 1994; Durrant et al, 1999; Maxwell-Armstrong et al, 1999). These patients demonstrate T-cell responses to antigen-positive tumours, infiltration of activated CD4 and CD56 lymphocytes and a significant increase in tumour cell apoptosis compared to unimmunized patients. Administration of this vaccine to over 170 patients with colorectal cancer has resulted in no discernible normal tissue toxicity, suggesting that the level of expression on tumour cells marks these cells as targets, leaving normal cells unaffected.

Colorectal and gastric tumours over-express CD55 that can be targeted by antibody and cell-mediated immunotherapy. The environment of the tumour also results in upregulation of endothelial cell surface-associated CD55 and an increase in the ability of these cells to secrete CD55 into their extracellular matrix. This may make CD55 a target on both tumour cells and the endothelium associated with the tumour.

### ACKNOWLEDGEMENTS

The skilled technical assistance of Mr R Moss is gratefully acknowledged. These studies were supported by the Cancer Research Campaign grant no sp220/0501.

### REFERENCES

Armitage NC, Perkins AC, Pimm MV, Farrands PA, Baldwin RW and Hardcastle JD (1984) Localization of an anti-tumour monoclonal antibody (791T/36) in gastrointestinal tumours. *Br J Surg* **71**: 407–412

Armitage NC, Perkins AC, Pimm MV, Wastie ML and Baldwin RW (1985) Imaging of primary and metastatic colorectal cancer using In-111-labelled anti-tumour monoclonal antibody 791T/36. *Nucl Med Com* **6**: 623–631

Austin EB, Robins RA, Durrant LG, Price MR and Baldwin RW (1989) Human monoclonal anti-idiotypic antibody to the tumour associated antibody 791T/36. *Immunol* **67**: 525–530

Buckley DJ, Chapman MA, Durrant LG, Robins RA and Armitage NC (1996) Increased expression of 791t-gp72 predicts a poor-prognosis in colorectal-cancer. *Gastroenterol* **110**: A 497–A 497

Collard CD, Vakeva A, Bukusoglu C, Zund G, Sperati CJ, Colgan SP and Stahl GL (1997) Reoxygenation of hypoxic human umbilical vein endothelial cells activates the classic complement pathway. *Circulation*, **96**: 326–333.

Coyne KE, Hall SE and Thompson ES (1992) Mapping of epitopes, glycosylation sites and complement regulatory protein domains in human decay accelerating factor. *J of Immunol* **149**: 2906–2913

Denton GWL, Durrant LG, Hardcastle JD, Austin EB, Sewell HF and Robins RA (1994) Clinical outcome of colorectal-cancer patients treated with human monoclonal antiidiotypic antibody. *Int J Cancer*, **57**: 10–14

Durrant LG, Buckley TJD, Denton GWL., Hardcastle JD, Sewell HF and Robins RA (1994) Enhanced cell mediated tumour killing in patients immunised with human monoclonal anti-idiotypic antibody 105AD7. *Cancer Res* **54**: 4837–4840

Durrant LG, Armstrong CM, Buckley D, Amin S, Robins RA, Carmichael JC and Scholefield JS (2000) A neoadjuvant clinical trial in colorectal cancer patients of the human anti-idiotypic antibody 105AD7, which mimics CD55. *Clin Cancer Res* **16**: 422–430

Durrant LG, Spendlove I, Buckley DJ, and Robins RA (2000) 105AD7 cancer vaccine stimulates anti-tumour helper and cytotoxic T-cell responses in colorectal cancer patients but repeated immunisations are required to maintain these responses. *Int J Cancer*, **85**: 87–93

Embleton MJ, Gunn B, Byers VS and Baldwin RW (1981). Anti-tumour reactions of monoclonal antibody against a human osteogenic sarcoma cell line. *Br J Cancer* **43**: 582–587

Farrands PA, Perkins AC, Pimm MV, Hardy JD, Embleton MJ, Baldwin RW and Hardcastle JD (1982) Radioimmunodetection of human colorectal cancers by an anti-tumour monoclonal antibody. *Lancet* **2**: 397–400

Farrands PA, Perkins A, Sully L, Hopkins JS, Pimm MV, Baldwin RW and Hardcastle JD (1983). Localisation of human osteosarcomas by anti-tumour monoclonal antibody. *J Bone and Joint Surg*, **65**: 638–640

Hamann J, Vogel B, Vanschijndel GMW and Vanlier RAW (1996) The 7-span transmembrane receptor CD97 has a cellular ligand (CD55, DAF). *J Exp Med* **184**: 1185–1189

Hatanaka M, Seya T, Matsumoto M, Hara T, Nonaka M, Inoue N, Takeda J and Shimizu A (1996) Mechanisms by which the surface expression of the glycosylphosphatidylinositol anchored complement regulatory proteins decay accelerating factor (CD55) and CD59 is lost in human leukaemia cell lines. *Biochem J* **314**: 969–976

Hindmarsh EJ and Marks RM (1998) Decay-accelerating factor is a component of subendothelial extracellular matrix in vitro, and is augmented by activation of endothelial protein kinase C. *Eur J Immunol* **28**: 1052–62

Jaffe EA, Nachman RL, Becker CG and Mindi CR (1972) Culture of human endothelial cell derived from umbilical veins: identification by morphological and immunological criteria. *J Clin Invest* **52**: 2745–2775

Lanzavecchia A, Lezzi G and Viola A (1999) From TCR engagement to T cell activation: A kinetic view of T cell behaviour. *Cell* **96**: 1–4

Maxwell-Armstrong CA, Durrant LG, Robins RA, Galvin AM, Scholefield JH and Hardcastle JD (1999) Increased activation of lymphocytes infiltrating primary colorectal cancers following immunisation with the anti-idiotype monoclonal antibody 105AD7. *Gut* **45**: 593–599

Nasu J, Mizuno M, Uesu T, Takeuchi K, Inaba T, Ohya S, Kawada M, Shimo K, Okada H, Fujita T and Tsuji T (1998) Cytokine-stimulated release of decay-accelerating factor (DAF;CD55) from HT-29 human intestinal epithelial cells. *Clin Exp Immunol* **113**: 379–85

Nicholson-Weller A and Wang C (1994) Structure and function of decay-accelerating factor CD55. *J Lab and Clin Med* **123**: 485–491

Niehans GA, Cherwitz DL, Staley NA, Knapp DJ and Dalmasso AP (1996) Human carcinomas variably express the complement inhibitory proteins CD46 (Membrane Cofactor Protein), CD55 (Decay-Accelerating Factor), and CD59 (Protectin). *Am J Pathol* **149**: 129–142

Sparrow RL and Mckenzie IFC (1983) HU-LY-M5 a unique antigen physically associated with HLA molecules. *Human Immunol* **7**: 1–15

Spendlove I, Li L, Carmichael J and Durrant LG (1999) Decay accelerating factor (CD55): A target for cancer vaccines? *Cancer Res* **59**: 2282–2286

Spring FA, Judson PA, Daniels G. et al (1987) A human glycoprotein that carries Cromer related blood group antigens on erythrocytes and is also expressed on leukocytes and platelets. *Immunol* **62**: 307–312

Tate CG, Uchikawa M, Tanner MJA, Judson PA, Parsons SF, Mallinson G and Anstee DJ (1989) Studies on the defect which causes absence of decay accelerating factor (DAF) from the peripheral blood cells of an individual with the inab phenotype. *Biochem J* **261**: 489–493

© 2001 Cancer Research Campaign

Tosello AC, Mary F, Amiot M, Bernard A and Mary D (1998) Activation of T cells via CD55: recruitment of early components of the CD3-TCR pathway is required for IL-2 secretion. *J Inflamm* **48**: 13–27

Vierboom MPM, Nijman HW, Offringa R, vanderVoort EIH, vanHall T, VandenBroek L, Fleuren GJ, Kenemans P, Kast WM and Melief CJM (1997) Tumor eradication by wild-type p53-specific cytotoxic T lymphocytes. *J Exp Med* **186**: 695–704

Vonderheide RH, Hahn WC, Schultze JL and Nadler LM (1999) The telomerase catalytic subunit is a widely expressed tumour-associated antigen recognised by cytotoxic T lymphocytes. *Immunity* **10**: 673–679

*© 2001 Cancer Research Campaign*
